# Cementless Short-Stem Total Hip Arthroplasty in Patients Aged 75 Years and Older: A Retrospective Single-Surgeon Study

**DOI:** 10.7759/cureus.93961

**Published:** 2025-10-06

**Authors:** Hanno Steckel, Helena Steckel

**Affiliations:** 1 Orthopaedics, MVZ Vitalis, Berlin, DEU; 2 Orthopaedics, University of Göttingen, Göttingen, DEU; 3 Medical School, University of Bonn, Bonn, DEU

**Keywords:** cementless total hip arthroplasty, elderly patients, implant survival, orthopedic surgery, short-stem implant

## Abstract

Introduction

Cementless short-stem total hip arthroplasty (THA) has gained popularity due to its bone-preserving potential and favorable biomechanical properties. However, its use in elderly patients remains controversial due to concerns about bone quality and implant stability. This study aimed to evaluate the safety and short- to mid-term outcomes of cementless short-stem THA in patients aged 75 years and older.

Methods

In this retrospective single-surgeon cohort study, 121 cementless short-stem THAs were performed in 117 patients (60% female, n=70; 40% male, n=47) between 2016 and 2024. All patients were aged ≥75 years (range, 75-90 years; mean, 78.7 years). Procedures were conducted via an anterolateral approach in the supine position.

Patient comorbidities were classified according to the American Society of Anesthesiologists (ASA) physical status classification system as ASA I (21.5%, n=26), ASA II (62.8%, n=76), and ASA III (15.7%, n=19), with a mean body mass index (BMI) of 28.5 kg/m². The mean operative time was 57 minutes, and the average hospital stay was 7.6 days. Patients were followed for a mean of 29.5 months (range, 2-110 months).

Results

At final follow-up, four complications (3.3%) were observed: three revision surgeries and one dislocation. The implant survival rate was 98%. No cases of aseptic loosening were recorded. Overall, the complication and revision rates were low and comparable to outcomes reported in younger patient cohorts.

Conclusion

Cementless short-stem THA appears to be a safe and effective option for patients aged 75 years and older, demonstrating excellent short- to mid-term outcomes with low complication and revision rates. These findings support the use of cementless short-stem implants in the elderly population with appropriate surgical technique and perioperative management.

## Introduction

Total hip arthroplasty (THA) is among the most successful orthopaedic procedures, providing significant pain relief and functional improvement. As life expectancy rises, the number of elderly patients undergoing THA continues to grow. Cemented stems have traditionally been preferred in this population due to concerns about bone quality and implant fixation. However, cementless short-stem designs have gained increasing acceptance for their bone-preserving characteristics, favourable load transfer, and ease of revision [1-5].

While excellent outcomes have been reported for cementless short stems in younger and middle-aged patients [6-8], their use in the elderly remains debated. Age-related osteoporosis, compromised bone quality, and higher comorbidity rates raise concerns about early loosening, periprosthetic fractures, and other complications [9]. Nevertheless, emerging evidence indicates that with appropriate patient selection and surgical technique, cementless short stems may provide outcomes comparable to or better than conventional stems in older adults [10,11].

Data specifically focusing on patients aged ≥75 years are limited. Most existing studies include heterogeneous age groups, leaving a gap in the evidence regarding the safety and efficacy of these implants in the elderly [12].

The aim of this retrospective study was to evaluate short- to mid-term clinical outcomes, complication rates, and implant survivorship of cementless short-stem THA in patients aged 75 years and older, all treated by a single surgeon using a standardized surgical approach.

## Materials and methods

This retrospective observational study was conducted between 2016 and 2024. A total of 121 cementless short-stem THAs were performed in 117 patients aged 75 years or older. Institutional review board (IRB) approval was obtained, and all patients provided written informed consent prior to participation. The preoperative Harris Hip Score (HHS) was recorded. Femoral morphology was assessed preoperatively in all cases using the Dorr classification system [13].

Patient selection

Inclusion criteria comprised patients aged ≥75 years undergoing primary THA for primary osteoarthritis. Exclusion criteria were prior hip surgery and the presence of significant femoral deformities. Baseline characteristics including age, sex, body mass index (BMI), and American Society of Anesthesiologists (ASA) physical status classification were documented.

Surgical technique

All procedures were performed by a single experienced surgeon using a standardized technique. Patients were positioned supine, and an anterolateral approach was employed, specifically the Ortho Center Munich (OCM) approach as described by Röttinger, modified for the supine position [14]. A cementless calcar-guided short stem (Optimys; Mathys Ltd., Bettlach, Switzerland) and a cementless press-fit acetabular component (Anexys; Mathys Ltd.) were implanted in all cases.

**Figure 1:**
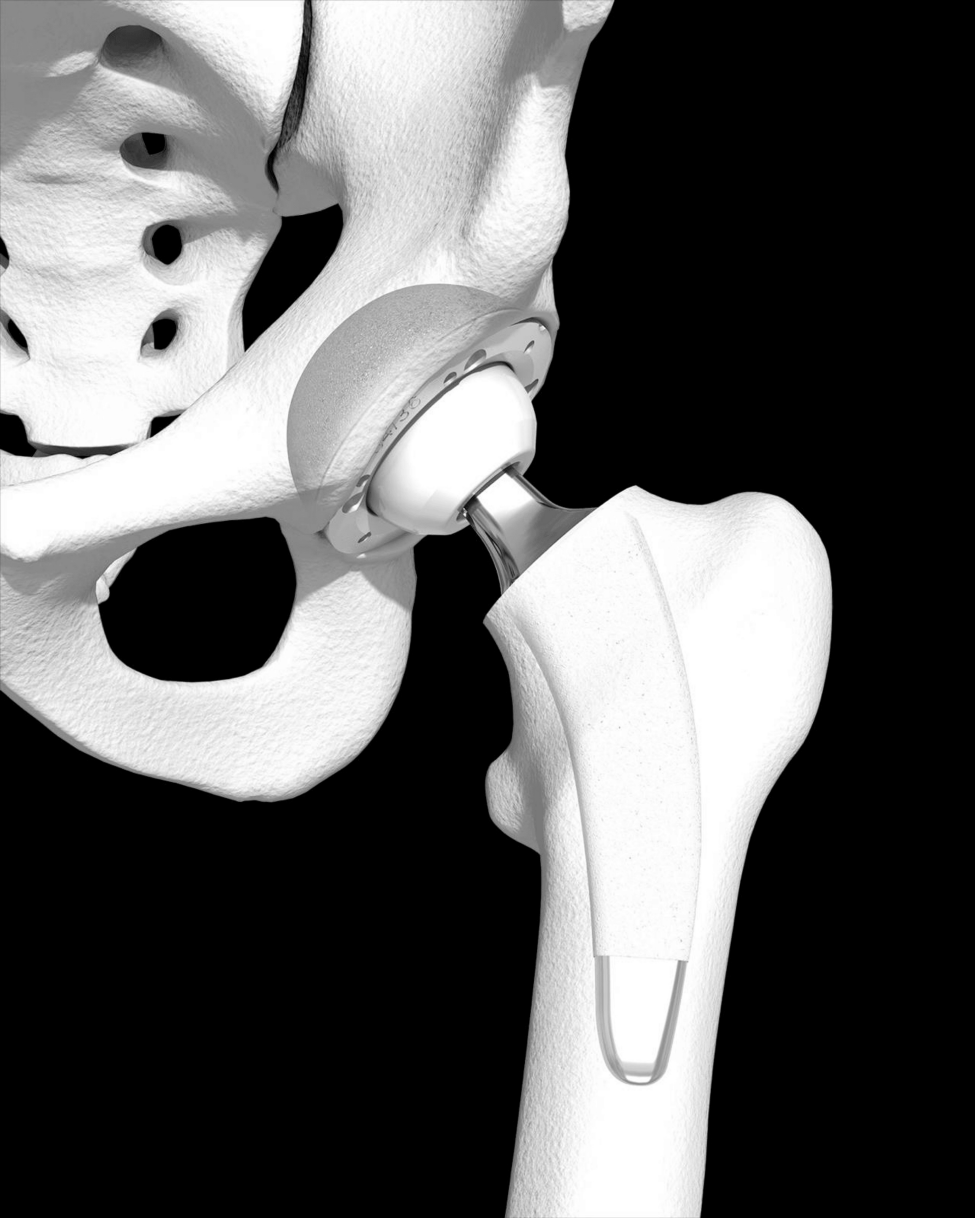
The Optimys short stem (Mathys Ltd., Bettlach, Switzerland) Republished with permission from Mathys Ltd. [15].

The Optimys stem, available in 12 sizes and two offset options, features a titanium plasma spray with calcium phosphate coating to facilitate metaphyseal fixation. The implant is designed for calcar and medial cortex alignment and achieves stability via the fit-and-fill principle and a three-point fixation system (Figure 1).

Perioperative management included a single intravenous dose of 2 grams of tranexamic acid to reduce blood loss and 1500 mg of cefuroxime for antibiotic prophylaxis. Standardized postoperative protocols were followed, including early mobilization and weight-bearing as tolerated.

Follow-up and outcome assessment

Postoperative clinical and radiographic evaluations were conducted at routine intervals. Clinical assessment included the HHS, documented at the one-year follow-up, if reached. Radiographic follow-up involved standardized anteroposterior pelvic radiographs, analyzed for evidence of implant loosening. Loosening was defined as progressive radiolucent lines ≥2 mm, component migration, or stem subsidence. Complications and revision procedures were documented. Patients who did not return for follow-up after discharge from hospital or rehabilitation were classified as lost to follow-up.

## Results

Clinical and radiographic outcomes

A total of 121 cementless short-stem THAs were performed in 117 patients (70 females, 60%; 47 males, 40%) with a mean age of 78.7 years (range, 75-90 years). The mean BMI was 28.5 kg/m². The distribution of the ASA physical status classification was as follows: ASA I in 21.5% (n=26) of patients, ASA II in 62.8% (n=76), and ASA III in 15.7% (n=19). The mean operative time was 57 minutes, and the average length of hospital stay was 7.6 days.

The mean follow-up duration was 29.5 months (range, 2-110 months), with four patients (3.4%) lost to follow-up. The mean preoperative HHS was 45.2 points. Among the 101 patients (83.5%) who completed the one-year follow-up, the mean HHS had improved to 88.4 points.

Preoperative femoral morphology, assessed using the Dorr classification, was categorized as type A in 16 patients (13.2%), type B in 99 patients (81.8%), and type C in six patients (5.0%).

Complications and revisions

A total of four complications (3.3%) were observed during the follow-up period.

Hematoma

One patient developed a postoperative hematoma requiring surgical intervention during the initial hospital stay. A single-stage revision was performed with exchange of the femoral head and liner. The postoperative course was uneventful. It was later revealed that the patient had continued clopidogrel therapy despite instructions to discontinue it prior to surgery.

Low-Grade Infection

**Figure 2:**
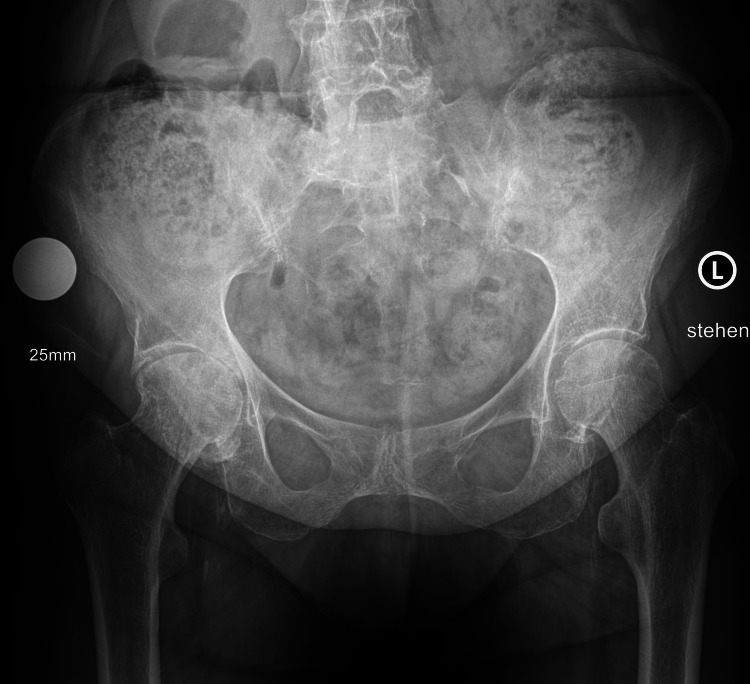
Preoperative radiographs before implantation

**Figure 3:**
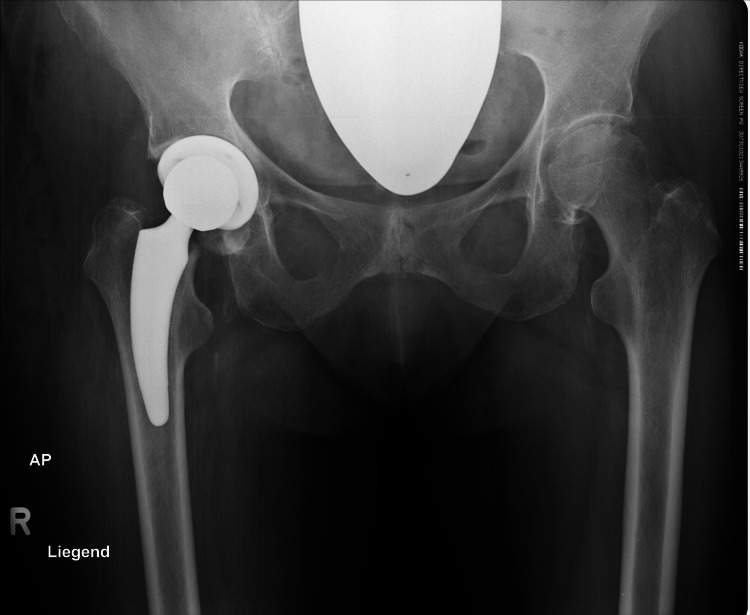
Postoperative radiographs after implantation of an Optimys stem (Mathys Ltd., Bettlach, Switzerland)

**Figure 4:**
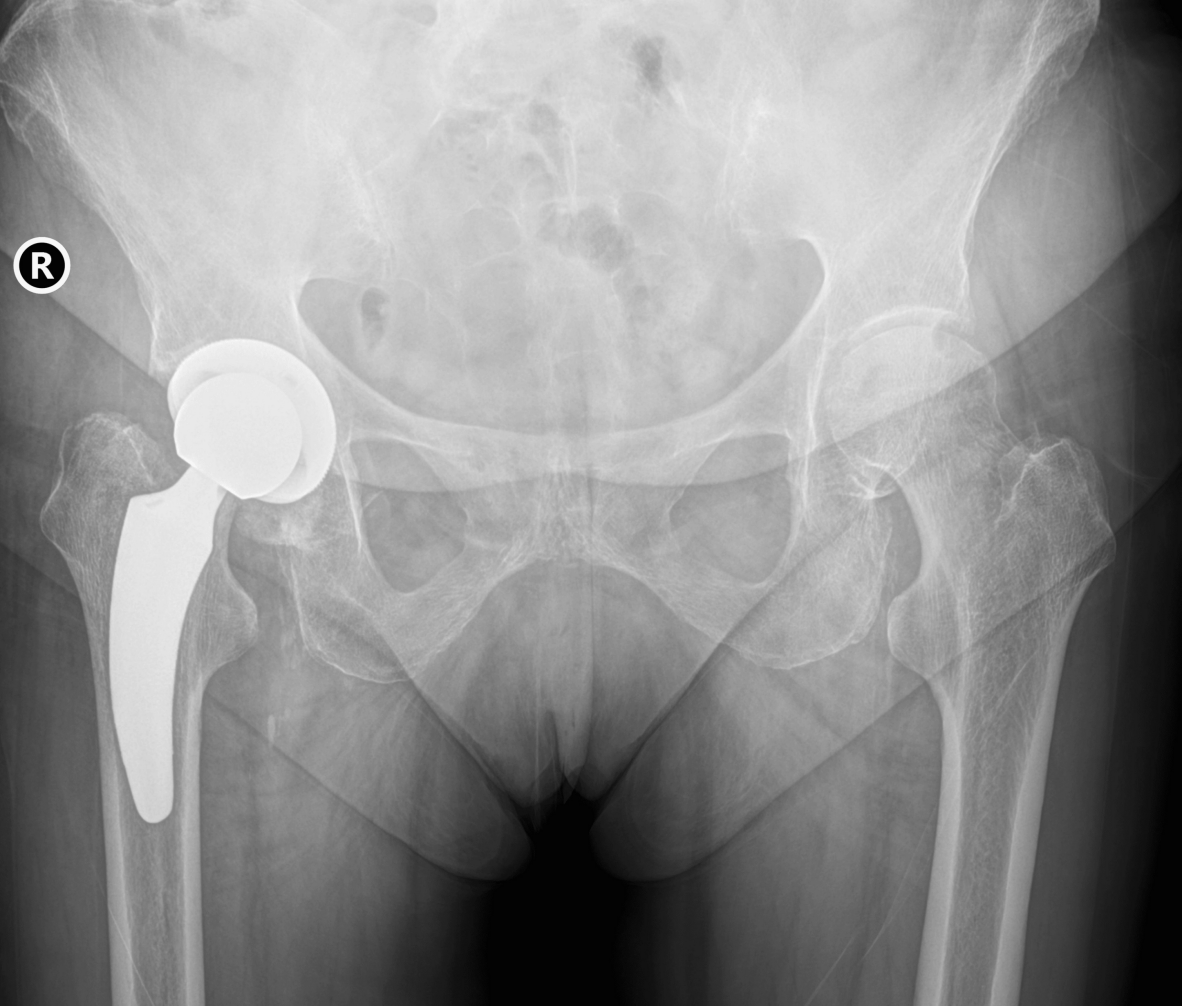
Postoperative radiograph with subsequent subsidence

**Figure 5:**
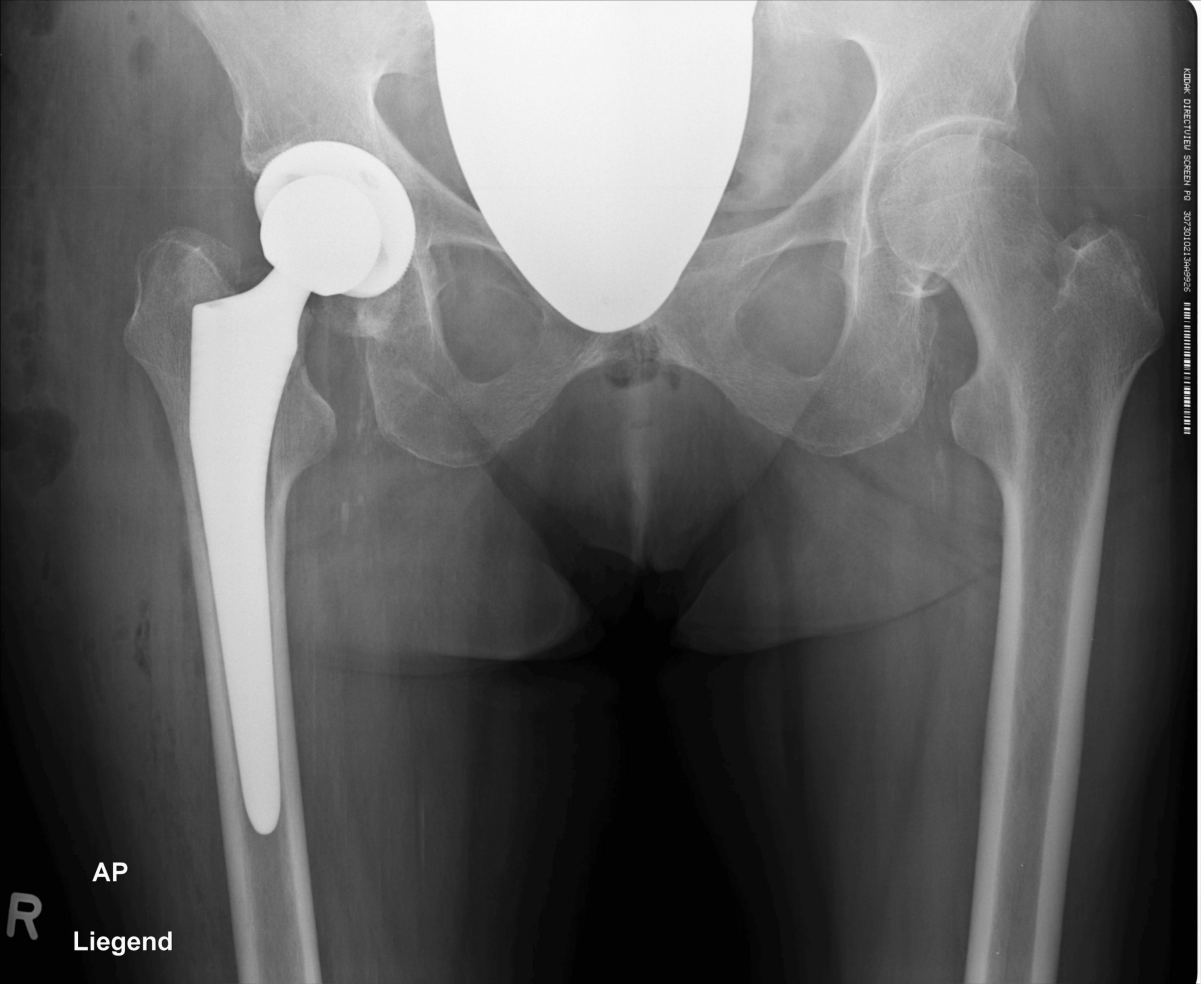
Revision with implantation of a twinSys stem (Mathys Ltd., Bettlach, Switzerland)

One patient presented postoperatively with stem subsidence, diagnosed at the rehabilitation facility. Intraoperative cultures confirmed a low-grade infection with Staphylococcus epidermidis. A single-stage revision with implantation of a standard straight stem (twinSys; Mathys Ltd.) was successfully performed (Figures 2-5).

Early Periprosthetic Joint Infection

One patient developed an acute deep infection during the index hospitalization, necessitating a single-stage revision with reimplantation of a new Optimys short stem.

Dislocation

One dislocation occurred and was successfully managed with closed reduction. No surgical revision was necessary.

All three revision cases remained free of further complications or reoperations at the most recent follow-up. No cases of aseptic loosening were observed. The overall implant survival rate, using revision of either the femoral or acetabular component for any reason as the endpoint, was 98.35% at final follow-up. No radiographic signs of implant loosening (i.e., progressive radiolucency ≥2 mm, migration, or subsidence) were detected (Tables 1, 2).

**Table 1:** Patient Demographics and Clinical Outcomes ASA: American Society of Anesthesiologists, BMI: body mass index

Parameter	Value
Number of hips	121
Number of patients	117
Female (%)	73 (60.3%)
Male (%)	48 (39.7%)
Mean age (years)	78.7
Age range (years)	75–90
Mean BMI (kg/m²)	28.5
ASA I (%)	26 (21.5%)
ASA II (%)	76 (62.8%)
ASA III (%)	19 (15.7%)
Mean operative time (minutes)	57
Mean hospital stay (days)	7.6
Mean follow-up (months)	29.5
Follow-up range (months)	2–110
Patients lost to follow-up (%)	4 (3.3%)
Complications	4 (3.3%)
- Hematoma requiring revision	1
- Low-grade infection with revision	1
- Early deep infection with revision	1
- Dislocation (closed reduction)	1
Aseptic Implant loosening	0%
Implant survival rate (%)	98.35%

**Table 2:** Distribution of Femur Morphology (Dorr Classification)

Dorr Type	Number of Patients	Percentage
A	16	13.2%
B	99	81.8%
C	6	5.0%

## Discussion

Historically, cemented conventional stems have been the preferred option in elderly patients due to concerns about osteoporotic bone and the need for reliable implant fixation [16-19]. However, cemented fixation is not without disadvantages. It has been associated with cardiovascular complications, including embolic events, particularly in frail patients [20,21]. Moreover, revision surgery of cemented stems can be technically demanding and carries risks such as periprosthetic fractures, incomplete cement removal, and cortical perforation [22,23].

In contrast, cementless short-stem THA offers several potential advantages, including a less invasive surgical approach, reduced operative time, and lower intraoperative blood loss [24,25]. Recent studies have reported promising outcomes for cementless short-stem THA even in elderly populations. Biomechanical analyses indicate that reliable metaphyseal fixation can be achieved with proper implant sizing and positioning - even in patients with compromised bone quality, such as Dorr type B or C femoral morphology [26,27].

The aim of this retrospective study is to contribute to the growing body of evidence by analyzing clinical and radiographic outcomes of 121 cementless short-stem THAs performed by a single experienced surgeon in patients aged 75 years and older.

At one year postoperatively, the HHS improved significantly from a preoperative mean of 45.2 to 88.4. This is particularly noteworthy given the advanced age and comorbidity burden of the cohort. The ASA physical status classification revealed that only 21.5% (n=26) of patients were ASA I, while 62.8% (n=76) were ASA II, and 15.7% (n=19) were ASA III. The mean BMI was 28.5 kg/m², placing the cohort in the WHO-defined pre-obese range.

A total of 121 THAs were performed in 117 patients, of whom 60.3% (n=70) were female, reflecting the typical sex distribution in this age group. The mean age at surgery was 78.7 years.

The mean operative time was 57 minutes - typically under one hour - a benchmark that is rarely achieved with cemented techniques. This reduced surgical duration may represent a clinical advantage in multimorbid elderly patients. The average hospital stay was 7.6 days, consistent with institutional standards aiming for a one-week inpatient course.

The mean follow-up duration was 29.5 months (range, 2-110 months), representing short- to mid-term outcomes, with a subset of patients followed for up to nine years. Preoperative femoral morphology was predominantly Dorr type B (81.8%, n=99), with only 5.0% (n=6) classified as Dorr type C, consistent with findings by Gkagkalis et al. [28].

Complication rates were low. Four complications (3.3%) were recorded: one hematoma requiring revision surgery, one dislocation managed conservatively, and two cases requiring stem revision - one to another short stem and the other to a standard straight stem. This results in an overall stem survival rate of 98.35% at final follow-up, with stem revision for any reason as the endpoint. These results are comparable to reported survival rates for both cemented and cementless stems [29,30].

No radiographic signs of aseptic loosening or early stem migration were observed, supporting the reliability of metaphyseal anchorage even in elderly patients. These findings align with the multicenter study by Gkagkalis et al., which reported a 98% survival rate and no cases of aseptic loosening in patients over 75 undergoing cementless short-stem THA [28]. Importantly, no postoperative periprosthetic fractures occurred, despite the presence of Dorr type C morphology in 5% (n=6) of patients. 

This study has several limitations. Its retrospective design, single-surgeon setting, and the use of only one specific short-stem implant may limit generalizability. Although the mean follow-up duration of 29.5 months permits mid-term assessment, longer-term data are needed to fully establish the durability of these implants in elderly patients.

Future research should focus on randomized controlled trials directly comparing cementless short-stem and cemented standard stems specifically for patients aged ≥75 years to provide age-specific safety profiles, ideally with follow-up periods beyond five to 10 years. Additionally, the inclusion of functional outcome parameters such as gait analysis and patient-reported outcome measures (PROMs) would further enhance understanding of the potential advantages of short-stem THA in this growing patient demographic.

## Conclusions

Our findings support the feasibility and safety of cementless short-stem THA in patients aged 75 years and older. The procedure was associated with excellent early implant survival, a low rate of complications and revisions, and no radiographic signs of aseptic loosening. When combined with thorough preoperative assessment, careful patient selection - including caution in patients with pronounced Dorr type C femoral morphology - and a standardized surgical technique, cementless short-stem THA represents a reliable and effective treatment option in the elderly. These results contribute to the growing body of evidence suggesting that cementless fixation, once considered less suitable in this population, may in fact offer distinct advantages when applied in a controlled and experienced setting.
